# Expanding the Diversity of *Actinobacterial Tectiviridae*: A Novel Genus from *Microbacterium*

**DOI:** 10.3390/v17010113

**Published:** 2025-01-15

**Authors:** Jacqueline M. Washington, Holly Basta, Angela Bryanne De Jesus, Madison G. Bendele, Steven G. Cresawn, Emily K. Ginser

**Affiliations:** 1 Department of Biology and Chemistry, Alliance University, New York, NY 10004, USA; amd4008@med.cornell.edu; 2 Department of Biology, Empire State University, Saratoga Springs, NY 12866, USA; 3 Department of Biology, Rocky Mountain College, Billings, MT 59102, USA; holly.basta@rocky.edu; 4 Weil Cornell Medicine, New York, NY 10021, USA; 5 Department of Biology, James Madison University, Harrisonburg, VA 22807, USA; bendelmg@dukes.jmu.edu (M.G.B.); cresawsg@jmu.edu (S.G.C.); 6 Biological Sciences Department, University of Pittsburgh, Pittsburgh, PA 15260, USA; emg190@pitt.edu

**Keywords:** *Microbacterium*, tectivirus, actinobacteria, jelly-roll capsid

## Abstract

Six novel *Microbacterium* phages belonging to the *Tectiviridae* family were isolated using *Microbacterium testaceum* as a host. Phages MuffinTheCat, Badulia, DesireeRose, Bee17, SCoupsA, and LuzDeMundo were purified from environmental samples by students participating in the Science Education Alliance Phage Hunters Advancing Genomics and Evolutionary Science (SEA-PHAGES) program at Alliance University, New York. The phages have linear dsDNA genomes 15,438–15,636 bp with 112–120 bp inverted terminal repeats. Transmission electron microscopy (TEM) imaging analysis revealed that the six novel phages have six-sided icosahedral double-layered capsids with an internal lipid membrane that occasionally forms protruding nanotubules. Annotation analysis determined that the novel *Microbacterium* phages all have 32–34 protein-coding genes and no tRNAs. Like other *Tectiviridae*, the phage genomes are arranged into two segments and include three highly conserved family genes that encode a DNA polymerase, double jelly-roll major capsid protein, and packaging ATPase. Although the novel bacteriophages have 91.6 to 97.5% nucleotide sequence similarity to each other, they are at most 58% similar to previously characterized *Tectiviridae* genera. Consequently, these novel *Microbacterium* phages expand the diversity of the *Tectiviridae* family, and we propose they form the sixth genus, *Zetatectivirus*.

## 1. Introduction

*Microbacterium* spp. are high G + C%, gram-positive rod-shaped aerobes belonging to the order *Actinomycetales* found throughout the environment in air, food, soil, plants, and water [1,2,3,4]. They are often non-harmful or even beneficial to plants [1,5]. Once thought to be rare in humans, *Microbacterium* infections have been identified with increasing frequency in patients [6]. *Microbacterium* spp. have been associated with bacteremia in immunosuppressed patients [7,8,9,10] and were even isolated from the sputum of a patient with cystic fibrosis, leading to speculation that the species could be an emerging pathogen of cystic fibrosis patients [11,12]. Although CRISPR-Cas9 evolved naturally as a defense mechanism in bacteria and archaea, it has been reported that *Microbacterium* spp. do not use CRISPR-Cas9 systems [13,14]. Other host defense mechanisms such as alternative restriction modification systems [15,16] have been identified.

*Microbacterium testaceum* is an endophytic bacterium that can be found in potato, rice, corn, and other agriculturally important crops [17]. Many gram-negative plant pathogens produce N-acylhomoserine lactone (AHL), a quorum sensing signaling compound, and strains of *M. testaceum* have shown early promise as a biocontrol agent with the ability to quench AHL-dependent quorum sensing by plant pathogens [17].

A diverse group of phages infect the *Microbacterium* spp. The genomes of these phages range from 15 kb to almost 195 kb, typically with high GC content ranging from 50.1% to 71.4%, which is expected given their high GC content host (average 67%) (https://phagesdb.org (accessed on 15 November 2024)) [1]. The list of phages that infect *Microbacterium* spp. is growing quickly; the first *Microbacterium* spp. phage was discovered in 2007 [18] and at current count, there are 5752 distinct phages that infect *Microbacterium* spp., most of which have a lytic lifestyle [1]. Of these, 11 phages have been isolated using *M. testaceum* as a host (https://phagesdb.org).

Over 26,000 bacteriophages have been isolated using bacterial hosts belonging to the order *Actinomycetales* by students participating in the Science Education Alliance—Phage Hunters Advancing Genomics and Evolutionary Science (SEA-PHAGES) program, an inclusive research community (iREC) with programmatic and scientific support provided by Howard Hughes Medical Institute (HHMI), the University of Pittsburgh, and James Madison University [19]. As course-embedded research projects, undergraduate students isolate, name, and characterize novel bacteriophages mostly from soil samples [19,20,21]. Phages infecting bacterial hosts within the phylum *Actinobacteria* are genetically highly diverse, but the vast majority belong to the class *Caudoviricetes* having double-stranded DNA genomes, isometric capsids, and tails. Of these, most have siphoviral morphology with long, flexible non-contractile tails [1]. Very few podoviral bacteriophages have been isolated which have short, non-contractile tails. Even rarer numbers of tailless phages have been isolated and therefore do not belong to the class *Caudoviricetes*.

The *Tectiviridae* family is a diverse group of non-enveloped, tailless DNA phages belonging to the class *Tectiliviricetes* with both lytic and lysogenic members, including the well-characterized *Enterobacteria* phage PRD1 [22]. Their genomes are linear, double-stranded DNA approximately 15 kb in length [23]. Hallmarks of the *Tectiviridae* family genomes include approximately 100 bp inverted terminal repeats (ITRs) [24] that flank around 30 open reading frames, including three conserved genes that encode (1) a DNA polymerase, (2) a double jelly-roll major capsid protein, and (3) a packaging ATPase [23,25,26,27]. *Tectiviridae* replicate their genomes using a protein-primed, strand-displacement mechanism [28] and a family B DNA polymerase [29]. The major capsid protein forms around a lipid membrane acquired from the host during assembly, creating an internal lipid membrane. Upon receptor binding on a new cell, this lipid membrane forms a proteo-lipidic tube, which allows passage of the phage genome into the host cell [30,31]. During assembly, an empty capsid forms, into which the phage genome is injected by a FtsK-HerA packaging ATPase integrated at one vertex [31].

*Tectiviridae* are structurally similar to several eukaryotic and archaeal viruses [32]. PRD1 is very similar to adenoviruses, members of the family *Adenoviridae*, which are non-enveloped, linear, double-stranded DNA tailless viruses that typically cause respiratory illnesses in vertebrates and belong to the class *Tectiliviricetes* (https://ictv.global/taxonomy). Adenoviruses are best known for their potential as vectors and were used as SARS-CoV-2 vaccine vehicles [33]. Both viruses have similar major coat proteins and capsid architecture in addition to linear genomes with inverted terminal repeats [34]. *Tectiviridae* are also very similar to archaeaviruses. Due to the similarity, the *Skuldviridae,* a new family of archaeaviruses was placed in the class *Tectiliviricetes* (https://ictv.global/taxonomy) [35]. The similarity of tectiviruses to both eukaryotic and archaeal viruses suggests the viruses may have a common ancestor, and tectiviruses may have played a very important role evolutionarily as the precursors of large eukaryotic transposons known as Polintons which are thought to have evolved into most eukaryotic dsDNA viruses [36,37].

Currently, the *Tectiviridae* family includes five genera and members (and proposed members) that infect both gram-positive and gram-negative bacteria. Tectivirus hosts include bacteria from the phyla *Pseudomonatoda* (infected by *Alpha-* and *Gammatectivirus*), *Firmicutes* (infected by *Betatectivirus*), and *Actinobacteria* (infected by *Deltatectivirus* and *Epsilontectivirus*) (https://ictv.global/taxonomy) [26,27]. Most of the phages belonging to the *Betatectiviridae*, *Deltatectiviridae,* and *Epsilontectiviridae* that infect gram-positive hosts are temperate [26,27,38] and presumably have a lysogenic lifestyle.

While lipid membrane-containing phages were once thought to be rare [39,40], advances in isolation and characterization have elucidated the pivotal role of these phages in microbial ecology, playing roles in the food chain and as vehicles for gene transfer [41,42,43]. Yet, in relation to other types of phages, they remain underrepresented, potentially due to the historical use of chloroform in culture-based studies to prevent bacterial contamination [43]. Lipid membrane-containing bacteriophages are sensitive to chloroform; in fact, sensitivity to chloroform is typically used as the first indicator of a lipid membrane. Recent evidence suggests that they are more abundant in the environment than previously believed and understanding them will pave the way for new innovations and applications, as well as provide further insight into viral lineages and evolution [22].

This study presents the isolation and characterization of six rare new tailless *Tectiviridae* members isolated from host *M. testaceum* by undergraduate students at Alliance University, NY (formerly known as Nyack College) in the SEA-PHAGES program. These novel tailless phages make up a monophyletic group which we propose represents a new sixth genus of *Tectiviridae: Zetatectivirus*. Previously, all the *Microbacterium* phages identified were members of the class *Caudoviricetes* and these are the first reported tectivirus phages isolated and characterized from *Microbacterium* spp. With an average genome size of 15,498 bp, they are currently the smallest phages known to infect *Microbacterium* spp. (https://phagesdb.org (accessed on 15 November 2024)).

## 2. Materials and Methods

### 2.1. Bacterial Strains

All the bacterial strains used in the study were obtained from the Agricultural Research Service (https://nrrl.ncaur.usda.gov/) and grown in peptone-yeast extract-calcium (PYCa) medium supplemented with 0.1% dextrose at 28 °C.

### 2.2. Isolation and Purification of Phages

Soil samples were collected in the United States in Nyack, NY, Baldwin, NY, Haskell, NJ, and Seabrook, MD. To isolate the phage, the samples were washed with PYCa media (1.0 g yeast extract, 15 g peptone, 2.5 mL 40% dextrose, and 4.5 mL 1M CaCl_2_ per liter) supplemented with 0.1% dextrose and filtered through a 0.22 μm filter. The filtered soil extracts were inoculated with mixed cultures including late exponential phase *M. testaceum* NRRL B-59317 and incubated at 28 °C with shaking for 48 h. After incubation, the cultures were filtered, and the filtrates plated in PYCa top agar lawn with *M. testaceum*. The plates were incubated for 2 days at 28 °C, following which isolated phages were picked into phage buffer with glycerol (10 mM Tris-HCl [pH 7.5], 10 mM MgSO_4_, 1 mM CaCl_2_, 68 mM NaCl, and 10% glycerol) to plaque purify. Crude lysates were obtained after three rounds of purification as previously described [44,45,46].

### 2.3. Phage Amplification and Production of High Titer Lysates

Crude phage stocks were diluted and plated on a lawn of the bacterial host to produce plates with near-confluent lysis. Plates were flooded with 8 mL phage buffer, and incubated overnight at 4 °C. To purify, the lysates were harvested and filtered through a 0.22 μm filter.

### 2.4. DNA Sequencing, Annotation, and Sequence Analysis

DNA was isolated from a high titer lysate purified by using Promega Wizard DNA extraction kits (http://www.promega.com/) or phenol-chloroform extraction using Phase-Lock Gel (http://www.quantabio.com/) and resuspended in ddH_2_O. The phage genomes were sequenced at the Pittsburgh Bacteriophage Institute where sequencing libraries were prepared from double-stranded phage genomic DNA using NEB Ultra II FS Kits. These were run on an Illumina MiSeq using 150-cycle v3 Reagent Cartridges yielding 150-base single-end reads representing between 106- and 4572-fold coverage of each genome (Table 1). The raw reads were quality-controlled using Consed version 29 and assembled using Newbler version 2.9. The assemblies were checked for completeness, accuracy, orientation, and genomic termini as previously described [47]. Phage genomes were annotated as described previously [48] using DNA Master (http://cobamide2.bio.pitt.edu), PECAAN (v2021–2024) (https://discover.kbrinsgd.org), Glimmer 3.0 [49], GeneMark 2.5 [50], NCBI BLAST 2.7 and Conserved Domain Database at NCBI [51], Starterator (http://phages.wustl.edu/starterator/) [52], Aragorn version 1.2.38 [53], tRNA-ScanSE 2.0 [54], HHPred (databases: PDB mmCIF70, Pfam-A, and NCBI Conserved Domain databases) [55,56], SOSUI [57], TMHMM (http://www.cbs.dtu.dk/services/TMHMM/) [58], DeepTMHMM [59], DNAbind [60], and Phamerator using database Actinobacteriophage_2422 [61]. EMBOSS was used to verify the terminal repeat sequences initially identified by sequencing [62]. Pairwise average nucleotide identities (ANI) were calculated using the Sequence Demarcation tool [63]. Default parameters were used for all software. All complete genome sequences are publicly available at phagesdb.org and in GenBank. Raw sequence reads are available at the Sequence Read Archive (SRA) (Table 1).

### 2.5. Host Range Analysis

High titer lysates were diluted tenfold in phage buffer with glycerol and 3 μL of each dilution was spotted on lawns of *Microbacterium* spp (*M. testaceum* NRRL B-59317, *M. arborescens* NRRL B-59342, *M. radiodurans* NRRL B-24799, *M. foliorum* NRRL B-24224, *M. saperdae* NRRL B-14833). Plates were incubated at 28 °C for 2 days and observed for plaque formation.

### 2.6. Electron Microscopy

High titer phage lysates were concentrated by centrifugation as described previously [44,45,46]. For negative staining analysis, phage particles were spotted onto formvar and carbon-coated 400 mesh copper grids, rinsed with distilled water, and stained with 1% uranyl acetate. Grids were imaged with a JEOL JEM 1400 transmission electron microscope at 100 kV acceleration voltage (JEOL, USA, Ltd., Peabody, MA, USA). Images were captured on an EMSIS Veleta side-mounted 2K × 2K CCD camera (Olympus-SIS, Munich, Germany).

### 2.7. Phylogenetic Reconstruction

Whole genome comparison was carried out using tectivirus complete genome nucleotide sequences retrieved from NCBI (accession numbers listed in Table 1) and Forthebois was included as an outgroup (MK620900.1). Tectivirus protein sequences (DNA polymerase, major capsid, and DNA packaging ATPase) were retrieved from NCBI Virus [64], omitting those sequences that have not been assigned a genus or classified as a particular protein. A T4 bacteriophage protein was included in each multiple alignment for an outgroup.

Within MEGA7 [65], MUSCLE [66] was used to construct multiple alignments of the complete genome nucleotide sequences using the following parameters: gap open penalty −400, gap extend penalty 0, max memory 4095 MB, max iterations 8, UPGMB clustering method, min diag length 24. This alignment was used to generate a UPGMA tree using the following parameters: bootstrap test of phylogeny (500 replicates) [67], amino acid substitution type nucleotide, maximum composite likelihood method, substitutions including transitions and transversions; rate of variation was calculated using a gamma distribution shape parameter = 1, homogeneous pattern among lineages with a complete deletion and including all codon positions. The tree was based on 13,980 positions.

MEGA7 [65] and MUSCLE [66] were used to construct multiple alignments of the DNA polymerase, major capsid, and DNA packaging ATPase protein sequences using the following parameters: gap open penalty −2.9, gap extend penalty 0, hydrophobicity multiplier 1.2, max memory in MB 4095, max iterations 8, clustering method UPGMB, minimum diag length 24. MUSCLE alignments were used to construct phylogenetic trees using the UPGMA method [68] using the following parameters: bootstrap test of phylogeny (500 replicates) [67], amino acid substitution using the Poisson correction method [69]; rate of variation was calculated using a gamma distribution shape parameter = 1, complete deletion. The DNA polymerase tree was based on 418 positions, the major capsid tree was based on 276 positions, and DNA packaging ATPase tree was based on 150 sites. All positions containing gaps and missing data were eliminated. Clades were collapsed by genus. Sequences found in each condensed clade can be found in Table 2.

### 2.8. Genome Comparisons

Comparative genome maps were constructed using Phamerator [61] (https://phamerator.org (accessed on 1 October 2024)) to display blastn alignments or an Observable notebook (https://observablehq.com/@cresawn-labs/phamerator-map) to display tblastx alignments.

## 3. Results

### 3.1. Isolation and Characterization of Microbacterium testaceum Phages

**Figure 1:**
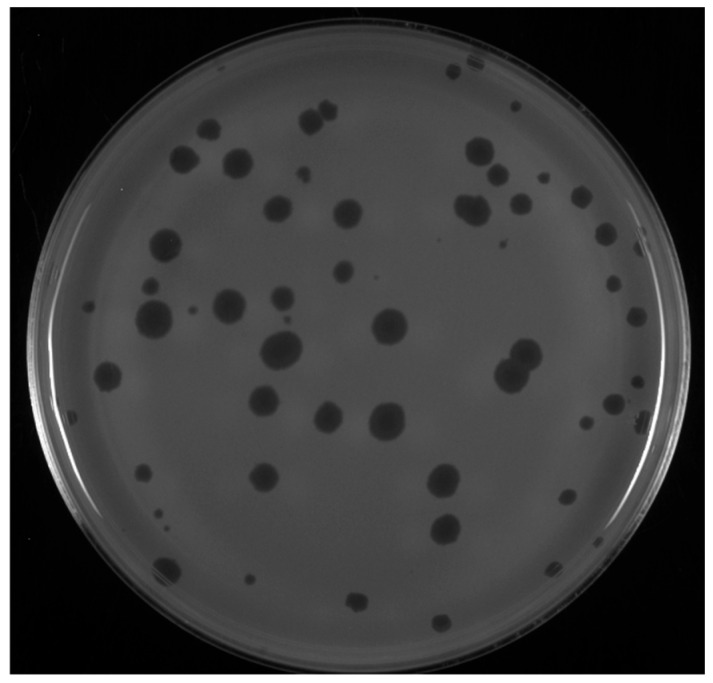
Representative plaques observed on a lawn of *M. testaceum*. A dilution of a purified lysate of SCoupsA was plated in a PYCa top agar bacterial lawn and grown for 48 h at 28 °C.

*Microbacterium* phages MuffinTheCat, Badulia, DesireeRose, Bee17, SCoupsA, and LuzDeMundo were isolated from environmental samples using host *M. testaceum* NRRL B-59317 by undergraduate students participating in the SEA-PHAGES program in 2019 and 2021. The phages were isolated from enriched soil samples collected at four discrete locations in the eastern United States (Table 1). After 48 h at 28 °C, they form 2–5 mm slightly turbid plaques on bacterial lawns of their isolation host (Figure 1).

### 3.2. Host Range Determination

**Figure 2:**
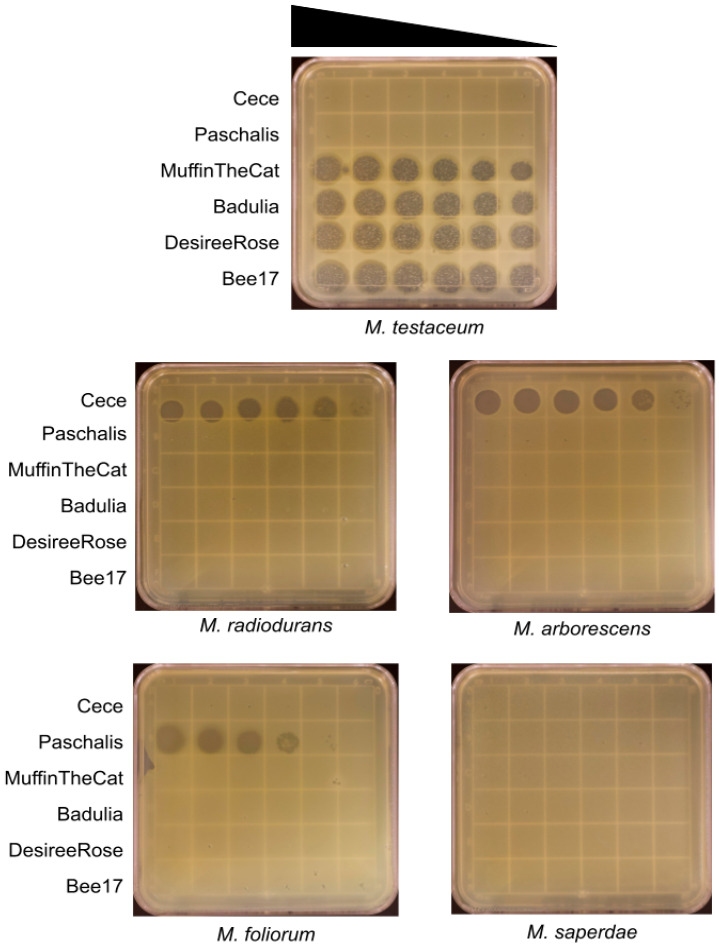
Host range analysis of novel tectiviruses. Tenfold dilutions of crude high titer lysate of each phage were spotted on lawns of *Microbacterium* spp. *Microbacterium* phages. Cece and Paschalis were isolated using *M. radiodurans* and *M. foliorum*, respectively, and were used as controls.

The host range of the novel *Microbacterium* phages was determined by spotting tenfold dilutions of high titer lysate on lawns of several *Microbacterium* spp. available in the laboratory including *M. arborescens* NRRL B-59342, *M. radiodurans* NRRL B-24799, *M. foliorum* NRRL B-24224, *M. saperdae* NRRL B-14833 in addition to the isolation host, *M. testaceum* NRRL B-59317 (Figure 2). Two sequenced *Microbacterium* phages with siphovirus-like morphology, Cece isolated using *M. radiodurans* NRRL B-24799 (Genbank Accession # OP068343) and Paschalis, isolated using *M. foliorum* NRRL B-24224 (Genbank Accession # MH155873) were used as controls. Host range analyses demonstrate that the novel *Microbacterium* phages have a narrow host specificity and only infect *M. testaceum* NRRL B-59317 (Figure 2). Although Cece and Paschalis were unable to infect *M. testaceum*, both infected their respective isolation hosts as expected. Interestingly, Cece also infects *M. arborescens* NRRL B-59342 with an efficiency of plating (EOP) of 100% (Figure 2).

### 3.3. Virion Visualization

**Figure 3:**
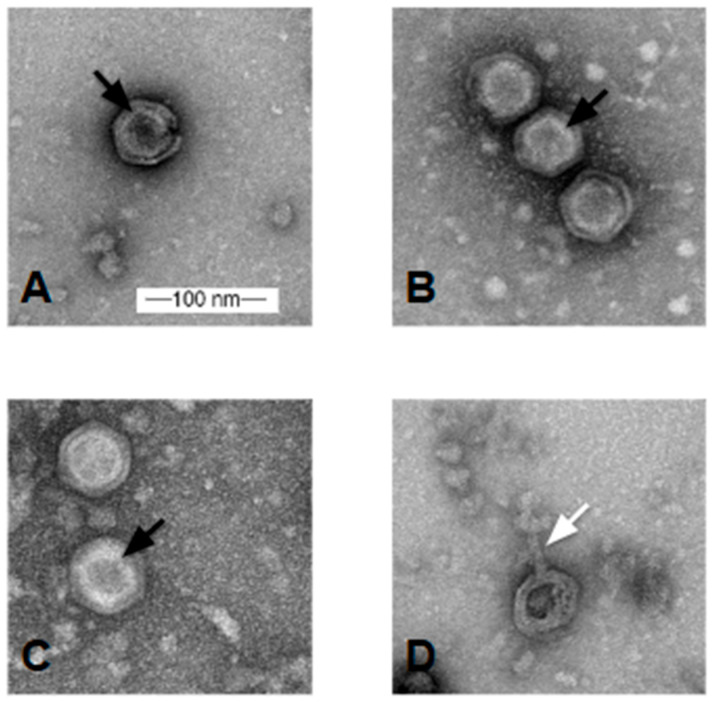
Transmission electron microscopy images of representative phages as observed after negative staining with uranyl acetate; images taken with JEOL JEM 1400 TEM at 100 kV acceleration voltage. (**A**) Bee17; (**B**) DesireeRose; (**C**) LuzDeMundo; (**D**) MuffinTheCat. Black arrow indicates the internal lipid membrane. White arrow indicates the tail tube.

Transmission electron microscopy (TEM) imaging analysis revealed that the novel *Microbacterium* phages have six-sided icosahedral double-layered capsids with a range of 57.8–66.3 nm and an average diameter of 60.6 nm (*n* = 14) (Bee17 *n* = 1; DesireeRose *n* = 3; LuzDeMundo *n* = 8; MuffinTheCat *n* = 2) (Figure 3). Several of the virion particles have nanotubes that protrude from a capsid vertex (Figure 3D, white arrow). Members of the *Tectiviridae* family are non-enveloped viruses that have double-layered capsids with an internal lipid membrane, and form protruding nanotubules at a low frequency [26]. Taken together, the TEM analysis of the novel *Microbacterium* phages suggests that they are new members of the *Tectiviridae* family.

### 3.4. Genome Sequencing

To genetically characterize these phages, genomic DNA was extracted from high-titer lysates, resuspended in ddH_2_O, and sequenced at the Pittsburgh Bacteriophage Institute. Sequencing was performed using an Illumina MiSeq Next Generation Sequencer with coverage ranging from 106 to 4572. Visualizing the assembled genomes revealed that the reads of all the genomes ended at a specific base without any circularizing reads. No finishing Sanger sequencing was required. The phage genomes are double-stranded and linear, ranging from 15,438 to 15,636 bp in length. All the genomes have 112–120 bp perfect ITRs (Table 1). Taken together, these data suggest that the phages are likely to have covalently bound terminal proteins and are similar in length to other members of the *Tectiviridae* family [70,71]. The GC content of the genomes ranges from 54.6 to 55.1% with an average of 54.9% (Table 1). The phage genome sequences are unlike any other actinobacteriophage sequence in PhagesDB (https://phagesdb.org/ (accessed on 15 November 2024)) and therefore, they are the founding members of the GE phage cluster.

### 3.5. Annotation

**Figure 4:**
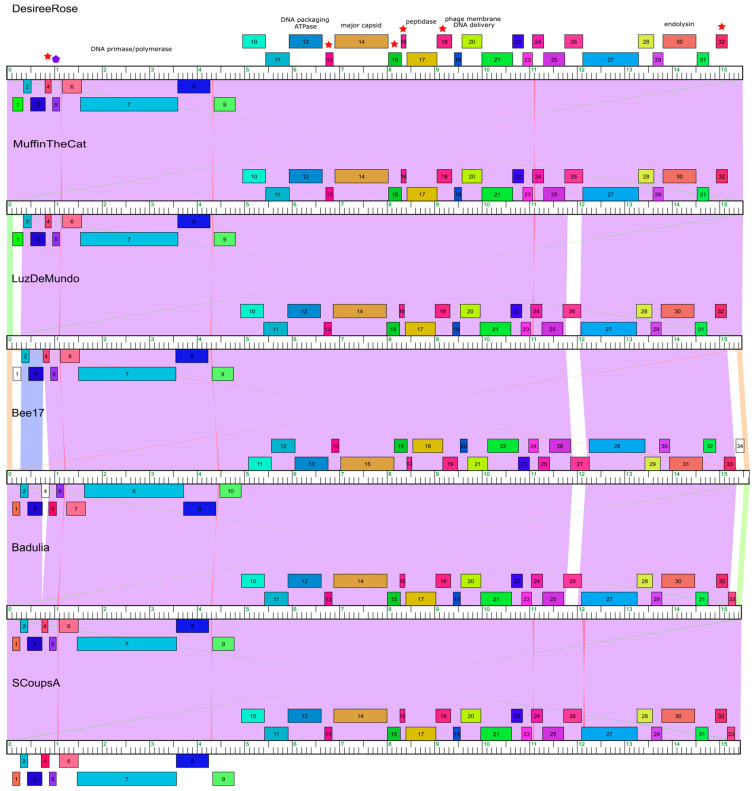
Phamerator genome maps of the six novel bacteriophages. The genomes are represented by horizontal marker bars, and the predicted ORFs are shown as numbered colored boxes either above (transcribed rightwards) or below (transcribed leftwards) the genome. LuzDeMundo has one orpham, gp1, a gene not closely related to any other known gene and Bee17 has two orphams, gp4 and gp34, indicated by the white boxes. The ORFs with predicted functions are labeled. Predicted transmembrane proteins are indicated by the red stars and DNA binding protein by the blue polygon. Purple indicates regions of high blastn similarity between phage genomes, whereas the white areas indicate sequences not shared across genomes. The other colored regions between genomes indicate areas with intermediate similarity.

Annotation analysis determined that the novel *Microbacterium* phages, MuffinTheCat, Badulia, DesireeRose, Bee17, SCoupsA, and LuzDeMundo all have 32–34 protein coding genes (Table 1, Figure 4). No tRNAs were identified using Aragorn version 1.2.38 [53] and tRNA-ScanSE 3.0 [54]. Similar to other members of the *Tectiviridae* family, the highly conserved DNA polymerase, DNA packaging ATPase, and major capsid genes were identified (Figure 4, Table 2). In addition, the function of three other genes could be ascertained with a high degree of probability using bioinformatic analysis: a peptidase, phage membrane DNA delivery protein, and endolysin. Six transmembrane membrane proteins and a DNA binding protein were also identified. The other 19–21 genes have an undetermined function (Figure 4, Table 2). All the genes in the genomes are homologs and there are no other members of each pham besides the six novel *Tectiviridae*. LuzDeMundo has one orpham, gp1, which is not present in any of the other novel phage genomes or related to any known gene, indicated by the numbered white box (Figure 4). Bee17 has two orphams, gp4 and gp34 (Figure 4). No integrase gene has been identified and it is unclear if the phages can form lysogens. Other tectiviruses, such as *Betatectivirus* pGIL01 and pGIL16, that form lysogens do not contain integrases and are maintained in the host as extrachromosomal replicating sequences [72].

**Table 2:** Assigned functions of genes in the genomes of the novel tectiviruses. The genes not included have undetermined functions.

Product Functions	Direction	Desiree Rose	Muffin TheCat	LuzDe Mundo	Bee17	Badulia	SCoupsA
membrane protein	R	gp4	gp4	gp4	gp5	gp4	gp4
DNA binding protein	R	gp5	gp5	gp5	gp6	gp5	gp5
DNA primase/polymerase	R	gp7	gp7	gp7	gp8	gp7	gp7
DNA packaging ATPase	F	gp12	gp12	gp12	gp13	gp12	gp12
membrane protein	F	gp13	gp13	gp13	gp14	gp13	gp13
major capsid protein	F	gp14	gp14	gp14	gp15	gp14	gp14
membrane protein	F	gp15	gp15	gp15	gp16	gp15	gp15
membrane protein	F	gp16	gp16	gp16	gp17	gp16	gp16
peptidase	F	gp17	gp17	gp17	gp18	gp17	gp17
membrane protein	F	gp18	gp18	gp18	gp19	gp18	gp18
phage membrane DNA delivery protein	F	gp19	gp19	gp19	gp20	gp19	gp19
endolysin	F	gp30	gp30	gp30	gp31	gp30	gp30
membrane protein	F	gp32	gp32	gp32	gp33	gp32	gp32

### 3.6. Genome Organization

**Figure 5:**
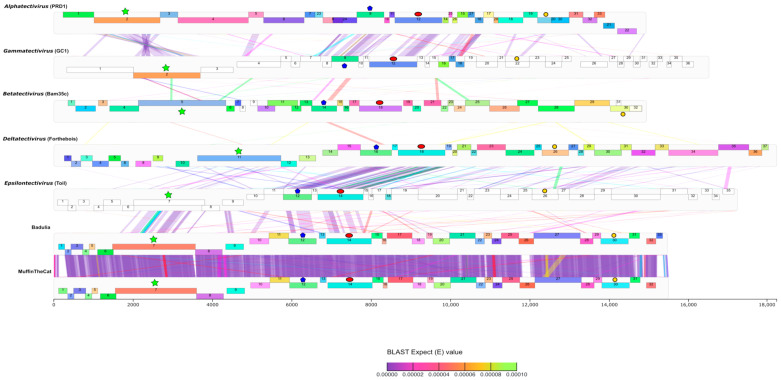
Genome maps of novel bacteriophages compared to representatives of each genus. The genomes are separated by gray borders, and the predicted ORFs are shown as numbered colored boxes either positioned towards the top of the gray border (transcribed rightward) or near the bottom of the gray border (transcribed leftwards). Genes are color-coded by phamily as in Figure 4. Gene phamilies with a single member are white. The colored regions between the genomes correspond to tblastx hits and are color-coded according to e-value. The DNA polymerase, DNA packaging ATPase, and major capsid are indicated by the green stars, blue polygons, and red ovals, respectively. The endolysin in each genome is indicated by the yellow circles.

Genome organization of the *Tectiviridae* family is highly conserved and it has been previously demonstrated that there is a family-wide conservation of three proteins: a major capsid protein, DNA packaging ATPase, and DNA polymerase [23,25,26,27]. The genomes of the six novel phages are organized with the first 9–10 genes on the left arm being leftwards transcribed with the remainder rightwards transcribed (Table 2, Figure 4). This two-segment genome organization is most similar to the *Deltatectivirus* (Wheeheim and Forthebois) and *Epsilontectivirus* (Toil) which are also tectivirus actinobacteriophages (Figure 5) [26,71]. The genome of *Gammatectivirus* (GC1) also has two segments with the first three genes on the left arm being leftwards transcribed (Figure 5). In the novel genomes, the highly conserved DNA polymerase is present on the left arm and transcribed leftwards (Figure 4 and Figure 5). The right arm contains structural genes and genes involved in cell lysis, such as the major capsid protein and endolysin (Figure 4 and Figure 5). Both genes are homologs of the PRD1 major capsid protein and a LysM-like endolysin, respectively. Also present on the right arm is a phage DNA delivery protein, a homolog of the PRD1 DNA delivery protein, which assists with delivery of DNA to the host (Figure 4) [23]. The similarity of the genome organization of the novel phages to the *Deltatectivirus* and *Epsilontectivirus* along with the presence of several homologs of the well-studied *Alphatectivirus* PRD1 supports the claim that they are tectiviruses.

### 3.7. Nucleotide Sequence Similarity

**Figure 6:**
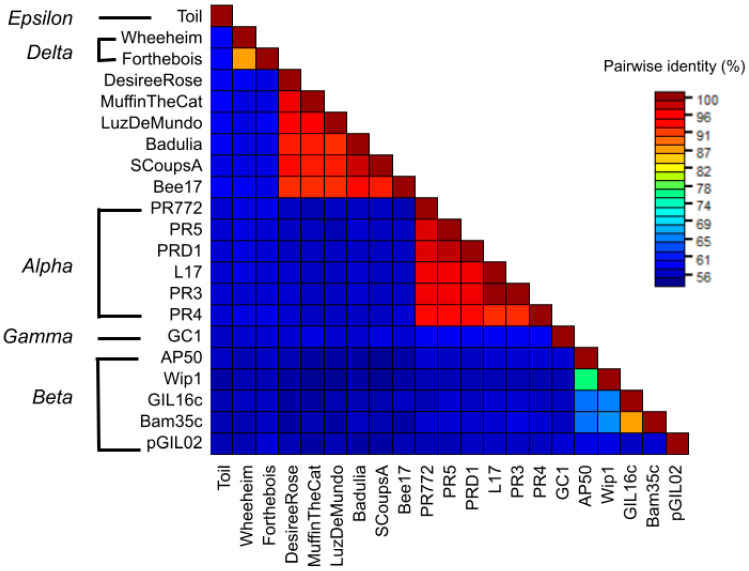
The average nucleotide identities calculated for the six novel *Microbacterium* phages and representative phages from each genus.

Whole genome comparison using average nucleotide identity (ANI) analysis is standard in species classification. This method is used by NCBI to evaluate the taxonomy of genomes submitted to GenBank and to determine relatedness between species [73]. Similarly, ANI can be used to determine virus taxon demarcation with a high degree of certainty [74]. ANI calculations show that the six novel bacteriophages have 91.6 to 97.5% nucleotide sequence similarity to each other (Figure 6). They are at most 58% similar to species within each of the current *Tectiviridae* genera. Therefore, the novel bacteriophages are most similar to each other and distinct from the other tectiviruses. This meets the criteria of these phages being members of a new species and a new genus separate from the known tectiviruses.

### 3.8. Sequence Analysis and Phylogenetic Reconstruction

**Figure 7:**
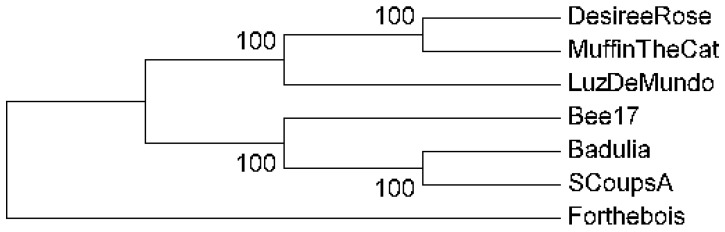
Phylogenetic reconstruction of novel tectiviruses complete genome nucleotide sequences. Trees were constructed using MEGA7 [65]. The UPGMA method [68] was used based on multiple alignments generated by MUSCLE [66]. Bootstrap values are based on 500 replicates and are indicated next to the branches [67]. First, second, third, and non-coding codon positions were included and all positions containing gaps and missing data were eliminated. See Table 1 for accession numbers. *Deltatectivirus* Forthebois was included as an outgroup (MK620900.1).

To determine the evolutionary history of the six novel phages, the full nucleotide genome sequences of Badulia, SCoupsA, Bee17, DesireeRose, LuzDeMundo, and MuffinTheCat were aligned and compared using phylogenetic reconstruction. Although these phages are closely related with over 90% pairwise nucleotide identity (Figure 6), they formed with high confidence two distinct clades: clade 1 contained DesireeRose, LuzDeMundo, and MuffinTheCat, while clade 2 contained Bee17, Badulia, and SCoupsA (Figure 7).

**Figure 8:**
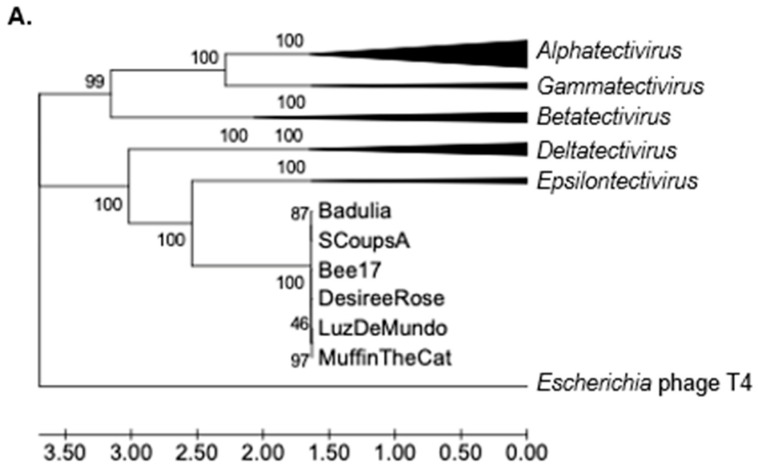

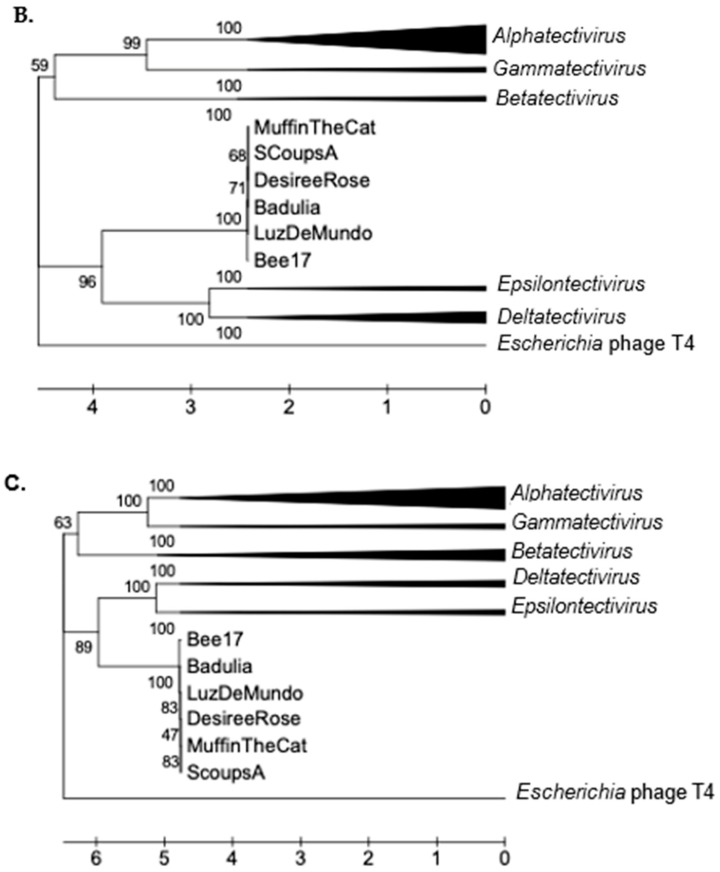
Phylogenetic reconstruction of tectiviruses (**A**) DNA polymerase, (**B**) major capsid, and (**C**) DNA packaging ATPase protein sequences. Trees were constructed using the UPGMA method [68] MEGA7 based on multiple alignments generated using MUSCLE [66]. Bootstrap values are based on 500 replicates and are indicated next to the branches [67]. Branch lengths indicate evolutionary distances, with units in amino acid substitutions per site, as computed using the Poisson correction method [69]. The rate variation was calculated using a gamma distribution shape parameter = 1. All positions containing gaps and missing data were eliminated. Clades were collapsed by genus. Sequences found in each condensed clade can be found in Table 3.

We further compared each of the three conserved proteins (DNA polymerase, major capsid, and packaging ATPase) of the six novel phages Badulia, SCoupsA, Bee17, DesireeRose, LuzDeMundo, and MuffinTheCat to previously characterized *Tectiviridae*, using multiple alignment of amino acid sequences (see Table 1 and Table 3 for accession numbers) and phylogenetic reconstruction (Figure 8A–C). Each of the three trees showed clustering of previously characterized tectiviruses into their expected genera (*Alpha*-, *Beta*-, *Delta*-, *Gamma*-, and *Epsilontectivirus*). The six novel phages, clustered together in a distinct clade for each of the three proteins. In each tree, this clade grouped most closely with *Delta*- and *Epsilontectivirus* which were also isolated from *Actinobacteria* species. As known clades were retained and bootstrap values were high, we have high confidence in the phylogenetic reconstruction. There is a clear delineation between the novel phages and the previously characterized genera, which, combined with the ANI data, suggests this clade should be considered a new genus.

## 4. Conclusions

This work demonstrates that based on viral morphology, genome size and organization, and the presence of the highly conserved DNA polymerase, major capsid, and DNA packaging ATPase, the novel *Microbacterium testaceum* phages MuffinTheCat, Badulia, DesireeRose, Bee17, SCoupsA, and LuzDeMundo are new members of the *Tectiviridae* family. Although these novel microbacteriophages are tectiviruses, they lack significant sequence similarity to other tectiviruses, and subsequent phylogenetic analysis revealed that they are distinct from phages in the five previously described genera and therefore, merits the formation of a sixth genus, *Zetatectivirus*. This expands not only the *Tectivirdae* family, but also the diversity of actinobacteriophages.

## Figures and Tables

**Table 1:** Phage discovery locations and general characterization of the novel tectiviruses.

Phage	GenBank Accession #	SRA Accession #	Yr Isolated	Location	Sequence Coverage	Genome Size (bp)	%GC	Terminal Repeats (bp)	# Genes
DesireeRose	OL455892	SRX27283292	2019	Nyack, NY, USA	4572	15,488	55	112	32
MuffinTheCat	MT952848	SRX27283295	2019	Nyack, NY, USA	824	15,494	55.1	114	32
LuzDeMundo	OP068334	SRX27283294	2021	Baldwin, NY, USA	537	15,471	55	113	32
Bee17	OQ709213	SRX27283290	2019	Haskell, NJ, USA	3019	15,636	54.6	119	34
Badulia	MZ150790	SRX27283289	2019	Nyack, NY, USA	4407	15,460	54.6	120	33
SCoupsA	OQ709206	SRX27283296	2021	Seabrook, MD, USA	106	15,438	54.8	120	33

**Table 3:** NCBI accession numbers for phylogenetic reconstruction, retrieved from NCBI Virus [64] and Genome Database (https://www.ncbi.nlm.nih.gov/genome/ (accessed on 31 January 2024)). The bacterial strain each phage was originally isolated from is listed; this does not represent its entire host range. When multiple strains have been sequenced, median GC content is listed. This is organized according to NCBI taxonomy browser: https://www.ncbi.nlm.nih.gov/Taxonomy/Browser/wwwtax.cgi?mode=Undef&id=10656&lvl=3&lin=f&keep=1&srchmode=1&unlock.

Subspecies	Phage (%GC)	Isolation Host (%GC)	DNA Polymerase	DNA Packaging ATPase	Major Capsid
*Alphatectivirus*					
*Alphatectivirus PR4*	PR4 (48.3)	*Pseudomonas aeruginosa* (66.2)	AAX45594.1, YP_337983.1	YP_337992.1, AAX45618.1	AAA32442.1, AAX45607.1, YP_337995.1
*Alphatectivirus PRD1*	L17 (48.3)	*Aeromonas hydrophila* (61.1)/*Escherichia coli* (50.6)	AAX45532.1	AAX45556.1	AAX45545.1
	PR3 (48.3)	*Pseudomonas aeruginosa* (66.2)	AAX45563.1	AAX45587.1	AAX45576.1
	PR5 (48.3)	*Escherichia coli* (50.4–50.8%)	AAX45625.1	AAX45649.1	
	PR772 (48.3)	*Proteus mirabilis* (38.8)	AAX45656.1, AAR99740.1	AAX45680.1 AAR99751.1 https://www.ncbi.nlm.nih.gov/nuccore/AY848688	AAX45669.1 AAR99754.1
	PRD1 (48.1)	*Pseudomonas* sp. (*P. aeruginosa* 66.2%)	AAA32450.1, AAA32452.1, AAX45903.1, YP_009639956.1	YP_009639965.1, AAX45556.1, AAX45649.1, AAX45680.1, AAX45927.1, AAR99751.1, P27381.3	UDY80299.1, AAA32445.1, AAX45916.1, YP_009639968.1, P22535.2
*Betatectivirus*					
*Betatectivirus AP50*	AP50 (38.7)	*Bacillus anthracis* (35.2)	YP_002302517.1	YP_002302526.1, ACB54913.1	YP_002302529.1
*Betatectivirus Bam35*	Bam35c (39.7)	*Bacillus thuringiensis* (34.9)	NP_943751.1	NP_943760.1	NP_943764.1
	pGIL02 (39.7)	*Bacillus thuringiensis* (34.9)	AND28856.1	Uncharacterized	AND28851.1
*Betatectivirus GIL16*	GIL16c (39.7)	*Bacillus thuringiensis* (34.9)	YP_224103.1	AAW33576.1, YP_224111.1	YP_224110.1
*Betatectivirus Wip1*	Wip1 (36.9)	*Bacillus anthracis* (35.2)	YP_008433325.1 *	Uncharacterized	AGT13371.1
*Deltatectivirus*					
*Deltatectivirus forthebois*	Forthebois (53.6)	*Streptomyces scabiei* (71.3)	YP_010084034.1, QBZ72843.1	QBZ72848.1	QBZ72850.1, YP_010084041.1
*Deltatectivirus wheeheim*	WheeHeim (54.6)	*Streptomyces scabiei* (71.3)	YP_010084070.1, QAX92919.1	QAX92924.1	QAX92926.1, YP_010084077.1
Unclassified	RickRoss (54.4)	*Streptomyces scabiei* (71.3)	UYL87501.1	UYL87506.1	UYL87508.1
*Epsilontectivirus*					
*Epsilontectivirus toil*	Toil (54.5)	*Rhodococcus opacus* (67.1)	YP_010084103.1, ARK07690.1	YP_010084108.1, ARK07695.1	(coat) ARK07697.1, YP_010084110.1
*Gammatectivirus*					
*Gammatectivirus GC1*	GC1 (50.5)	*Gluconobacter cerinus* (55.6)	YP_009620046.1, ATS92570.1	YP_009620053.1, ATS92577.1	ATS92580.1, YP_009620056.1
Outgroup					
	T4 (35.3)	*Escherichia coli* (50.6)	NP_049662.1	NP_049775.1	AAA32503.1

* There is an annotation error for Wip1; the accession number listed for the DNA polymerase is not the polymerase protein. Wip1 was therefore omitted from the DNA polymerase tree.

## Data Availability

All complete genome sequences are publicly available at phagesdb.org and in GenBank. Raw sequence reads are available at the Sequence Read Archive (see Table 1).
